# 2356: openSESAME: a “search engine” for discovering drug-disease connections by leveraging publicly available high-throughput experimental data

**DOI:** 10.1017/cts.2017.71

**Published:** 2018-05-10

**Authors:** Adam C. Gower, Avrum Spira, Marc E. Lenburg

## Abstract

OBJECTIVES/SPECIFIC AIMS: Microarray technology has produced large volumes of gene expression data profiling differences in gene expression in a vast array of conditions, much of which is publicly available. Methods to query these data for similarities in patterns of gene regulation are limited to comparisons between preannotated groups. In response, we developed openSESAME to find experiments where a set of genes is similarly coregulated without regard to experimental design. An important application of openSESAME is drug repositioning: if a pattern associated with disease is reversed by a given drug, the drug might target disease-related processes. METHODS/STUDY POPULATION: Experiments from the Gene Expression Omnibus (GEO) were normalized, signature-association (SA) scores computed for each sample, experiments assigned enrichment scores, and ANOVAs used to assign significance to experimental variables automatically extracted from GEO. SA scores were also generated for hundreds of publicly available signatures, and pairwise correlations used to create a relevance network. RESULTS/ANTICIPATED RESULTS: Using signatures of estrogen and p63, we recovered relevant experimental variables, and with the network approach, we recovered previously reported associations between disease states and/or drug treatments. DISCUSSION/SIGNIFICANCE OF IMPACT: openSESAME has the potential to illuminate “dark data” and discover novel relationships between drugs and diseases on the basis of common patterns of differential gene expression.

